# Participatory approach to flood disaster management in Thohoyandou

**DOI:** 10.4102/jamba.v11i3.711

**Published:** 2019-07-04

**Authors:** Ndidzulafhi I. Sinthumule, Ntavheleni V. Mudau

**Affiliations:** 1 Department of Ecology and Resource Management, School of Environmental Science, University of Venda, Thohoyandou, South Africa; 2 Department of Geography and Geo-Information Sciences, School of Environmental Science, University of Venda, Thohoyandou, South Africa

**Keywords:** Participatory Rural Appraisal, Participatory Approach, Disaster Management, Floods, Communities

## Abstract

In recent years, there has been a paradigm shift in research from ‘top-down’ directives to ‘bottom-up’ planning. Thus, there has been a change from imposing strategies to a participatory approach by indigenous people. This study uses the participatory approach to flood disaster management in Thohoyandou and its environs. The aim of this study is twofold: first, to understand the perception of communities towards floods hazards; and second, to probe how communities respond to flood hazards and how this knowledge can be used in the planning and management of future disasters. In order to achieve these objectives, participatory rural appraisal (PRA), interviews and observation were used as data collection techniques. The study found that there was consensus among the participants that flooding is a natural process, but human activities enhance the risks of flooding. Human activities that were found to be the causes of flood included clearance of vegetation, cultivation in steep slope areas, the effect of relief, urbanisation, poor designs and maintenance of drainage system and settlement in inadequate areas. The study found that local communities did not cope when there was flooding. However, they suggested strategies that should be used to cope with future flood hazards.

## Introduction

For centuries, the general worldwide history of flood disasters has been dominated by top-down approaches. This required no interaction with populations concerned; either solutions were implemented without consultation or populations were given directives as to how to reduce flood hazard impacts (Mercer et al. 2008). In the regions where flood disasters occurred, hard (structural) and soft (non-structural) approaches for flood disaster management were implemented (Yamada et al. 2011) without involving local population. However, it has increasingly been recognised that focusing upon structural (such as dams and levees) (Mercer et al. 2008; Yamada et al. 2011) and non-structural strategies such as improved land-use planning, relocation, flood proofing, flood forecasting and warning and insurance (Bradford et al. 2012; Petrow et al. 2006), and associated mitigation strategies without involving the communities has not been enough to prevent the ever-increasing impacts of flood hazards upon populations (Mercer et al. 2008). Over the past four decades, participatory approaches have been introduced as an alternative to flood disaster management (Chambers 1994; Fordham 1999; Khan & Rahman 2007; Mercer et al. 2008; Nunes Correia et al. 1998; Wehn et al. 2015). In other words, in order to enhance local flood disaster mitigation, participatory approaches for flood disaster management are proposed.

The concept of participation is rapidly becoming a catch-all concept, even a cliché. The term ‘participation’ has been interpreted in many ways (Pretty 1994), ranging from passive participation (where people are included in a project merely by being told about it) to self-mobilisation (where people take initiatives and responsibilities with or without limited external influence). In this study, participation can be considered as an act of sharing and contributing responsibilities based on consensus building. This implies that all those involved in the activity or responsibility are recognised to have something to contribute and, as a matter of fact, are prepared to accept any outcome as a result of their action or inaction (Dovie 2003). Thus, participation enables local people to seek their own solutions according to their priorities (Cornwall & Jewkes 1995). As Cornwall (2003) has noted, efforts to promote participation in projects, programmes and policy consultation appear to offer the prospect of giving a voice and a choice to everyone who has a stake. In development projects, participatory approach grew out of holding out the promise of inclusion of creating spaces for the less vocal and powerful (particularly the poorest of the poor) to exercise their voices and begin to gain more choices (Cornwall 2003; Michener 1998). The idea is to involve all stakeholders, including the government, local communities, non-governmental organisations (NGOs), media, the private sector, academia, neighbouring countries and donor communities (Khan & Rahman 2007).

Wehn et al. (1994) noted that the concept of participation by local communities in development projects has become important in the contemporary world and in some cases a pre-requisite for donors’ funded projects. In disaster management, the concept of participation is also gaining momentum because disasters are local events that primarily affect local communities. No one is therefore more interested in reducing flood disaster risk than those whose survival and well-being is at stake. Furthermore, as local people are those immediately affected when disasters occur, they become the first responders to the event (Gaillard & Mercer 2013). It therefore makes sense that local communities should be the prime participants of disaster management. This article contributes to recent debates over the use of participatory approaches by examining the use of participatory approach within flood disaster management. Two broad questions structure this article. Firstly, what are the perception and attitudes of communities towards flood hazards? Secondly, how do local communities respond to flood hazards? Knowledge on how local communities cope with flooding may help in the planning and management of future flood disasters. In working towards answering these research questions, the study uses Thohoyandou and its environs to demonstrate how local communities respond to flood hazards. The first section of this article presents a brief overview of participatory approach and its significance in the contemporary world. The second part explains the location of the study area and the methods used to collect and analyse data. The third section presents results and discussions, while the last section presents the conclusion.

## Participatory approach

Pain and Francis (2003) defined participatory approach as a technique that place emphasis on participants producing detailed accounts of a certain topic using their own words and frameworks of understanding. This approach arose as a result of the perceived limitations of the top-down approach through a promotion of participation and an involvement of local people (Mercer et al. 2008; Wisner et al. 2004). As Pain and Francis (2003) have noted, the defining characteristic of participatory research is not so much on the methods and techniques employed, but the degree of engagement of participants within and beyond the research encounter. Thus, the idea of participatory approach is to discover solutions to problems from participants or local communities through participatory techniques (Ivanitz 1999). The motivation for participation by local people is that beneficiary involvement makes projects more likely to succeed in meeting their objectives. In other words, if local people participate actively in project planning and implementation, they are more committed to its success (Michener 1998). Furthermore, Michener (1998) argued that participation facilitates local people’s acceptance of new policies and technologies promoted by outsiders. Through beneficiary participation, indigenous knowledge can be gained, and local labour, financial and in-kind contributions can lower the implementation costs. There is also a belief that participation rescues the development industry from being top-down, paternalistic and dependency-creating. This helps conscientious development practitioners feel better about their intervention, but also genuinely shifts the focus from the development professionals’ interests to the so-called beneficiaries (Michener 1998).

Pain and Francis (2003) noted that the diversity of participatory approaches is growing. These growing methods include Participatory Appraisal (PA) – where local people are involved at all stages (from priority setting to solution implementation) and emphasis is placed on education and collective action – as well as research and Participatory Rural Appraisal (PRA) or Participatory Learning and Action (PLA), which involves participatory diagramming with other techniques such as interviewing and observation. In addition, there is also Rapid Rural Appraisal (RRA) or rapid or Relaxed Appraisal (RA) – which is used where ownership of research lies with an external researcher – and participatory action research (PAR) – which is a form of action research that emphasises the participation of research subjects. Importantly, PAR rather than PA places more emphasis on the outcomes than on the value of the process itself (Dovie 2003; Pain & Francis 2003). Although a wealth of research on these participatory approaches has been used in the field of geography (Pain & Francis 2003), health research (Cornwall & Jewkes 1995; Khanlou & Peter 2005), anthropology (Hoffman & Oliver-Smith 2002), development studies (Michener 1998) and disaster risk reduction research (Khan & Rahman 2007; Mercer et al. 2008), their potential for use within flood disaster management research and subsequent development of strategies has not been fully investigated. In this study, the use of participatory techniques and their relevance for flood disaster management has been explored.

## Study area and methods

**FIGURE 1:**
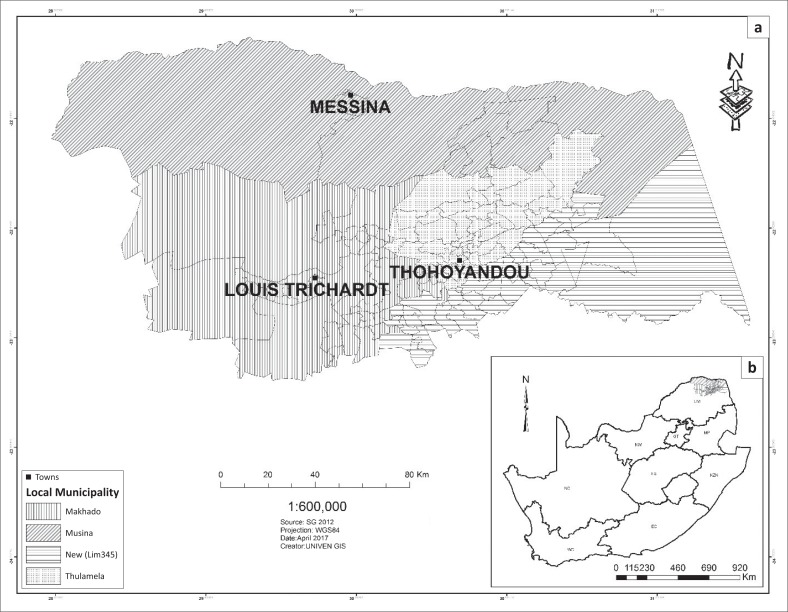
(a & b) Location of the study area.

Thohoyandou and its environs were affected by floods in 2000. Ten years later, the area was once again affected by severe floods. The enormous destruction caused by floods in Thohoyandou and the surrounding villages in 2000 and 2010 has led to this study. Thohoyandou is a town under Thulamela Municipality found in Vhembe region (Figure 1).

Thulamela Municipality is situated in the far north-eastern part of Limpopo province, South Africa, with a population of more than 600 000 individuals. The study area lies between latitudes 22˚ 15’ and 25˚ 45’ south and longitudes 29˚ 50’ and 30˚ 31’ east. To the east, Luvuvhu River forms a boundary with Kruger National Park in South Africa. Thohoyandou lies at an altitude of 240 m with a total area of 6677 km² (Rix et al. 1989). Thohoyandou is located within a subtropical climatic region with high temperatures and humidity in summer and mild winters. The study area has hot summer months and cool winter months. In addition, the area receives seasonal rainfall in summer. Thohoyandou receives average rainfall of 638 mm a year, much of this falling between October and March. The wettest months are December and January.

The aim of this study was to understand the perception of communities towards flood hazards and to probe how communities respond to flood hazards and how this knowledge can be used in the planning and management of future disasters. From the beginning, the emphasis has been to encourage participation of those who were affected by floods and engage them in suggesting and acting upon solutions that can be used in future and for policy-making. As we have noted, four participatory approaches have been identified in the literature (Dovie 2003; Pain & Francis 2003). In order to achieve the aim and research questions of this study, PRA method (Chambers 1994, 1997; Dovie 2003; Pain & Francis 2003) was found more suitable. This technique, now also known as PLA (Chambers 2002; Dovie 2003; Pain & Francis 2003), was found appropriate because it concentrates on exploring diversity in communities and allowing communities to design solutions to their own problems (Cronin et al. 2004). In addition, this methodology often involves participatory diagramming with other techniques such as interviewing and observation.

The fieldwork that support this study was undertaken in Ndondola village, Duthuni village, Maniini village, Makwarela Extension 3 and Thohoyandou Block F and G and Thohoyandou town, with local communities (52), traditional leaders (eight), business people (seven) and Thulamela and Vhembe officials (four) and government officials dealing with disaster management (two) in Thulamela Municipality. A variety of methods were used (including semi-structured interviews and observation), but the cornerstone of the study was participatory diagramming in small groups of four to six people. The participants aged between 25 and 50 years. Each group was provided with a large sheet of white papers, glue stick, scissors, coloured highlighters and coloured makers in order to discuss, sketch and write down their experiences regarding 2000 and 2010 floods. In order to maximise the effectiveness of participatory appraisal, participants were given a sequence of tools designed specifically for the project (Box 1). This was done in order to encourage groups to raise, discuss, expand on and prioritise the issues that mattered to them regarding floods, and to suggest and evaluate possible solutions. Interviews and observation were also conducted with participants in order to get clarity on some points drawn and raised during discussions.

**BOX 1:** Examples of participatory visual techniques used in this study.

1. Brainstorming – participants were asked to think about the word ‘floods’. Participants were requested to write down or draw their understanding of floods or identify any aspects associated with floods.
2. Timeline – participants were asked to draw a timeline of how floods had affected their life. Some people talked about their experiences as victim of floods, whereas others talked about incidences that had affected friends and relatives but had some impact on themselves. Identify what other organisation or government department that had been involved during flooding, and whether that involvement had been good or bad.
3. Cause or impact diagrams – participants were asked to identify the factors that have caused and exacerbated floods as well as the impacts of floods from their village perspective.
4. Ranking the biggest cause of floods – participants were asked to identify the biggest cause of flood in their area.
5. Coping mechanisms – participants were asked to explain or draw how they coped with recent floods. They were also requested to design solutions that can be used in future, based on their recent experiences.

In addition, interviews were also conducted with those stakeholders who were unable to attend the community participatory meetings. Purposive sampling was used to select members to be interviewed. The information written on large sheets of papers and notes taken during discussion by the researcher were analysed qualitatively. The main points emerging were extracted from the group work materials. Similarly, the main narrative that came from semi-structured interviews was also used to analyse the understanding and perception of communities towards flood hazards and how communities responded to flood hazards. The key points that arose from group discussions and interviews were verified through field observation in the affected areas. Other sources of data used in this study include newspaper articles published during floods in the study area.

### Ethical consideration

Permission to conduct this research was obtained from local municipalities, chiefs and headmen. Informants were interviewed based on their willingness and availability. Respondents were assured that their answers would be kept confidential; to implement this, the names of interviewees are not revealed in this paper.

## Results and discussion

The results of the two floods (2000 and 2010) obtained through PRA are presented in this section. The study found that there was consensus among participants that excessive rainfall led to flooding, and that flooding is a natural process that cannot be stopped. The studies conducted by Fordham (1992) and Nunes Correia et al. (1998) also found similar results. There was also consensus among participants that flooding is a threat to everyone. Although floods pose danger to people, it was agreed that human activities enhance the risks of flooding. Local communities have identified clearing of vegetation as one of the factors that exacerbated the risk of flooding in their area. Local communities cut trees to clear land for agriculture and for fuel-wood. This leaves the land unprotected, leading to excessive soil erosion. With no trees, water flows at a high speed with devastating effects on property. This finding is not unique to Thohoyandou; rather, it was also found in other areas (Clements 2009; Nunes Correia et al. 1998). Communities have suggested planting of trees within household as a strategy that should be adopted by everyone to reduce the speed of water flow during flooding. It is believed that this will lessen the effects of floods in future.

Other identified causes of floods include cultivation on steep slopes or mountainous ecosystems as well as the effect of relief. For instance, Ndondola village, Duthuni village, Makwarela Extension 3, Thohoyandou Block F and G and Thohoyandou town are all situated at the foot of the mountains, with Maniini village found on the southern side of Thohoyandou town on a very gentle slope. The study found that water from mountains came at a higher speed, which caused severe damages to human settlements at the foot of the mountain. These results are similar to the findings by Musyoki, Murungweni and Thifhulufhelwi (2016). In addition, some local communities in Thohoyandou Block F and G, Thohoyandou town and Makwarela Extension 3 were of the view that urbanisation also worsened floods by reducing the permeability of ground surfaces and increasing runoff rates, which is similar to the results found by Parker 1999 cited by Few (2003). In Thohoyandou town, concrete and tarmac used for road and pavement constructions were reported to have made the area impermeable, and as a result, high volume of water stagnated, resulting in devastating effects on infrastructure.

Local communities have also identified allocation of plots for settlement in inadequate areas (such as those in valleys, along rivers and in wetlands) as the main factor causing flood disaster in both rural and urban areas. Similar results have also been found by studies conducted in other areas (Adelekan 2010; Few 2003; Musyoki et al. 2016; Sanderson 2000; Smith 1996). In Ndondola and Duthuni villages on the western side of Thohoyandou town, some plots were found to be allocated on valleys and others on flood-prone areas. In these villages, communities who were staying in flood plain areas were found to be poorer with low level of education. The study found that the houses were of poor quality and the impacts were more severe when compared with the damages in other areas. These results confirm the findings of Canesio (2010) that households with lower educational attainment and annual income tended to be more vulnerable to the risks and threats from flooding. The communities in these villages reported that when there was no rainfall, they did not experience problems; however, when there was heavy rainfall or floods, they found themselves in danger, as stated clearly by two community members in Ndondola village:

I was given this plot in 2005 by the chief and I gladly accepted it. At first I did not know that I was building in a dangerous area because the land was dry. I also liked the area because it is closer to Thohoyandou and the soil is also fertile. I started to experience problem in 2010 when there was floods. My house was under water and all my belongings were affected by water. (Interview, community member 2013)

I bought this plot in 2007 and the area was dry. Initially I did not know that the owner sold the property because there was problem of water. It only came to my mind in 2010 when my house was under water that I bought an area that is not conducive for settlement. (Interview, community member 2013)

In Thohoyandou Block F and G, Thohoyandou town and Makwarela Extension 3, the study found that some businesses and residential plots were allocated in wetland areas. Although all wetlands that appear on the Thulamela municipal plan are demarcated as parks, it was startling to know that business and residential areas were also allocated to such land, which should have been considered only for setting up parks. Although some respondents knew that they were staying in a wetland or in waterlogged areas, they were of the opinion that with time water will disappear in the area. People did not experience problems before flooding occurred because the land was initially dry. However, in 2000 and in 2010, buildings were under water and some buildings developed cracks, whereas others simply collapsed because of high volumes of water stagnation. Essentially, it can be said that when there were heavy rains or floods, communities were unable to cope with the huge volume of water passing in the area, as stated clearly by one community member in Ndondola village:

This area is not suitable for human habitation. We are staying in a valley and the water coming from hills and mountains pass in this area. We are not only affected when there are floods, even the slightest rainfall has a huge impact on us. I regret the day I was given this plot because we live in fear in this area. (Interview, community member 2013)

Consequently, local communities temporarily move out from their homes to their relatives and neighbours when heavy rainfall or floods commence. The study found that some local communities were rescued by friends, relatives and neighbours who were on the spot at the time of floods. Other community members whose houses were under water were given temporary shelter in local schools and churches, whereas some were given tents by local authorities when there was heavy rainfall or floods. However, communities disliked this approach because it could only offer a short-term remedy and could not minimise future hazards. Some were unable to reach their homes when temporary bridges were swept away by water. In the same manner, others were unable to go to work or schools because of poor road conditions. Others became casualties of floods when their houses collapsed while they were inside. These results confirm the findings of other studies in Africa where flooding was a serious threat to communities (Adelekan 2010; Christie & Hanlon 2001; Motsholapheko, Kgathi & Vanderpost 2011; Sanderson 2000). In order for communities to cope with flooding in future, they were of the view that government should help them to build proper houses with firm foundation, particularly in rural areas. Other community members, particularly those who were in flood line areas, were of the view that if alternative land is available, they would prepare themselves to relocate with the help from local government. In other words, communities suggested relocation as an alternative strategy to avoid future disaster by floods. This is contrary to that reported by Rashid, Hunt and Haider (2007), who noted that despite extensive experience with flood problems, communities in Dhaka, Bangladesh did not consider relocating to flood-free areas as an option. Rather, they would prefer a reduction in the risk of flooding at their current location.

Some community members, particularly those in Thohoyandou Block F and G and Makwarela Extension 3, were concerned about development of potholes. This made difficult driving from homes to work when there was heavy rainfall or floods. Communities complained that some of them damaged their cars and did not receive any compensation from government because of too many potholes in roads during floods. In addition, poorly constructed roads and bridges, as well as the lack of storm water drainage, worsened floods in villages. This is similar to Adelekan (2010), who identified lack of storm water drainage as the major cause of flooding in Lagos, Nigeria. In the study area, participants felt that government should take full responsibility to help them and to build proper roads and bridges to ensure that there is free movement of water and free movement of people when there are floods. In addition, they suggested that local authorities should build storm water drainage to enhance free movement of water during floods.

Local authorities (particularly chiefs and headmen) stated that people who were desperate to get land for settlement would force them to allocate plots in flood-prone areas. For instance, in the case of Ndondola village near Thohoyandou, initially all the valleys were allocated for cultivation because it was known that the area was not suitable for human habitation. However, pressure from local communities who were desperate to get land for development forced local authorities to allocate plots for residential areas in valleys and flood-line areas. Although such areas face no problems when the land is dry, the settlements in this area become dangerous and risky during heavy rainfall or floods. Local authorities also suggested that local communities in flood-prone areas should be relocated to more suitable areas.

Business people in Thohoyandou whose structures were on wetlands were rich and had a high level of education. As a result, they were not worried about flood hazards. The business owners indicated that they were not affected by floods because during construction, the original wetland soil was removed and replaced by gravel sand, which was compacted by heavy machines to ensure that the ground would be strong. It was indicated that about 10 m of gravel was added in the area with the idea of elevating the foundation to ensure that water do not have any impact on the buildings. In other words, the foundation of the buildings that were constructed on the wetlands was made to be strong in such a way that water cannot penetrate and affect the buildings. Consequently, business owners in Thohoyandou were not affected by floods in 2000 and 2010. This is in support of the findings by Canesio (2010), who argued that households with higher educational attainment and annual income tended to be less vulnerable to the risks and threats from flooding.

In addition, the study also found that business people have insurance to protect their shops and businesses against any kind of disaster that may happen in the area. Although the shops of business people could cope with flood hazards, they were worried only about the drainage system in Thohoyandou. Upon heavy rainfall or flooding, drainage systems would become clogged and the release of water became slow, which makes the area to remain flooded throughout the rain or floods. This problem is attributed to poor design and lack of drainage system maintenance within Thohoyandou. Inadequate or poor urban drainage system has also been reported as the factor that caused floods in Setúbal, Portugal (Nunes Correia et al. 1998) and Lagos, Nigeria (Adelekan 2010). Business people are of the view that the local municipality should inspect the drainage system regularly to ensure that there is no water blockage to avoid flood-related damages in future.

Municipal officials were of the view that though wetlands were an important ecosystem, when there is a need to expand the town, they were forced to allocate plots for either residential or business purposes. They indicated that before allocation was made, an environmental impact assessment and a geotechnical study were conducted to check the suitability of the area. If the area was found suitable, it was rezoned as either residential or business area, and plots were allocated to those who needed them.

## Conclusion

The use of participatory approach to flood disaster management has helped to understand the viewpoint of local communities towards flood hazards, coping strategies and future strategies that can be used to minimise flood hazards. This critical information would have been impossible to obtain without involving those who bear the cost of flooding. In essence, the use of participatory approach in flood disaster management has offered opportunity for all interested and affected communities to voice their experiences and concern (Williams 2004). As local people are those immediately affected when floods occur, they become the first responders to the event and they are able to give a first-hand experience. Rather than only relying on strategies imposed by government or private companies as it has been the situation in other countries, this study has shown that flood disaster management requires local strategies coming from local communities. In other words, data gathered through participatory approach give a far more solid understanding of the realities faced by local population than by just using strategies imposed by local authorities or government, which in many cases is not conducive to an understanding of the locality and situation as a whole. Essentially, participatory approach promotes bottom-up planning as opposed to top-down approach, which has been criticised by social scientists for imposing strategies on local communities (Gaillard & Mercer 2013; Mercer et al. 2008; Wisner et al. 2004). The knowledge obtained from local communities through participatory approach (which includes allocation of plots for business and residential areas in unsuitable areas, and poor design and maintenance of drainage systems) can be used in the planning and management of future disasters. It can also be used to develop sound policies and legislations at local, provincial and regional levels. This article concludes that flood disaster management requires a participatory and an integrated approach involving all relevant parties within a community.
